# Integrative Learning of Disentangled Representations from Single-Cell RNA-Sequencing Datasets

**DOI:** 10.34133/csbj.0015

**Published:** 2026-03-16

**Authors:** Claudio Novella-Rausell, Dorien J. M. Peters, Ahmed Mahfouz

**Affiliations:** ^1^ Department of Human Genetics, Leiden University Medical Centre, 2333 ZA Leiden, the Netherlands.; ^2^ Leiden Computational Biology Center, Leiden University Medical Center, Leiden, the Netherlands.; ^3^ Delft Bioinformatics Lab, Delft University of Technology, Delft, the Netherlands.

## Abstract

Single-cell RNA sequencing enables comprehensive analysis of cellular diversity across biological systems. While current batch correction methods can jointly define cell types across multiple conditions, individuals, or modalities, they typically require matching features or paired samples across datasets. Here, we present shared-private Variational Inference via Product of Experts with Supervision (spVIPES), a probabilistic framework that decomposes unpaired single-cell datasets with nonmatching features into shared and private components. spVIPES learns a probabilistic latent variable model that separates dataset-specific (private) from conserved (shared) cellular features across groups. We implement both supervised and unsupervised variants: the supervised version uses cell-type annotations to guide the Product of Experts, while the unsupervised version leverages optimal transport to identify cell correspondences without requiring labels. We evaluate the performance of spVIPES using simulated data and demonstrate its utility across 3 diverse biological scenarios: (a) cross-species comparisons, (b) regeneration following long and short acute kidney injury, and (c) interferon-β stimulation of peripheral blood mononuclear cells. spVIPES effectively disentangles dataset-specific and conserved cellular features while matching or exceeding state-of-the-art methods for batch correction. Furthermore, spVIPES’ shared latent space enables more accurate cell-type identification across datasets with nonmatching features compared to existing methods. We implemented spVIPES using the scvi-tools framework and release it as an open-source software at https://github.com/nrclaudio/spVIPES.

## Graphical Abstract

**Figure:**
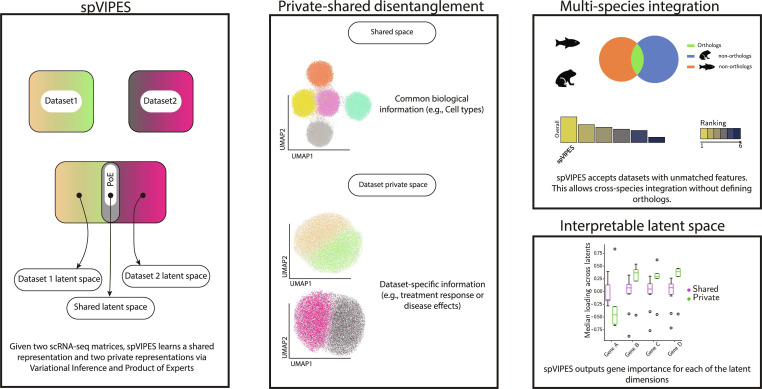

## Introduction

Advances in single-cell technologies allow the characterization of transcription variability across hundreds of thousands of cells collected from multiple biological samples. Modeling cells’ response to internal or external stimuli has greatly improved our understanding of disease, stimulation, or development [1–3]. Deep generative models have been widely used to model variation in single-cell data, offering a scalable solution to capture nonlinear variation [4–6]. For example, models such as scVI [6] have been applied to build “reference atlases” [7–9], which capture cell heterogeneity in a given organ or tissue while accounting for the variability between studies, individuals, or any other metadata at a larger scale. This is accomplished by jointly modeling the cells from the compendium as originating from a single set of latent variables through a uniform generative process.

Current variational autoencoders (VAEs) for single-cell data attempt to model variation using a uniform set of latent variables, posing a key limitation when analyzing cells from samples that belong to different groups (e.g., different treatments, perturbations, or species). In these complex datasets, VAEs attempt to push all sample-level variation into a common latent space, resulting in representations where phenotypic variability cannot be easily disentangled from nuanced differences between the groups. Representation disentanglement has proven useful in tasks such as cross-domain adaptation or multimodal learning in computer vision [10,11]. For example, DMVAE [12] disentangles the latent space of image-text pairs into “private” and “shared” representations. The shared representation is obtained through a Product of Experts (PoE) and captures domain-invariant information, like plant species and color. The private representations isolate domain-specific details, such as background, foreground, or shape for images, and smell or medical properties for text descriptions. The disentangled shared representation can be generalized across a more diverse set of samples, improving model performance.

The disentanglement of domain-specific variation is particularly valuable in biological contexts [13]. ContrastiveVI [14] obtains “shared” and “salient” variables specific to background and target scRNA-seq datasets, respectively. However, an explicit background–target relationship between the datasets is needed, which is nontrivial to define, for example, in multispecies comparisons. MultigroupVI [15] handles multiple groupings in a unified model that can disentangle “private” and “shared” sources of variation by training an inference network that learns a posterior distribution over all cells, regardless of group, together with as many extra inference networks as groups are present. scDisInFact [16] uses a set of shared and unshared encoders to disentangle multicondition and condition-specific factors of variation. Furthermore, while deep generative models utilizing Mixture of Experts [17] have been successful in single-cell multiomics integration, the potential of PoE frameworks for explicitly separating shared biological signals from group-specific variation in unpaired transcriptomic data remains underutilized.

In addition, current approaches employing VAEs often lack interpretability due to the nonlinearity of their mappings [18]. While recent methods such as linearly decoded VAE (LDVAE) [19], VAE enhanced by gene annotations (VEGA) [20], and siVAE [21] have introduced linear decoders or interpretable architectures to address this challenge, they do not inherently structure the latent space into shared and private components for multigroup analysis. Another limitation of current models is aligning features across datasets. As varying preprocessing strategies and aligning methods are used, the resulting datasets often have mismatched features. This is especially relevant when considering data from different species. The greater the evolutionary distance between species, the harder it is to identify 1-to-1 orthologs [22]. Recently, several methods have been developed to take advantage of RNA [23] or protein [24] sequence similarity to help match the features across species. Building on this, dedicated cross-species integration tools such as SATURN [25], TACTiCS [24], scPoli [26], and others [27,28] have emerged to address these feature mismatches.

Here, we present spVIPES (shared-private Variational Inference via Product of Experts with Supervision). We leverage VAEs and PoE to model groups of cells into a common explainable latent space and their respective private latent spaces. Using different encoders allows spVIPES to handle cells with unmatched features, while using linear decoders allows interpretation of all latent spaces. We demonstrate spVIPES’ ability to disentangle private and shared representations while accounting for batch effects by comparing shared and private space integration metrics with state-of-the-art linear and nonlinear methods across simulated and real-world datasets. We also demonstrate spVIPES’ performance in cell-type representation learning with largely nonmatching features by integrating distant species and comparing the quality of learned cell types with dedicated cross-species methods such as SATURN. Finally, we also show how spVIPES’ learned gene weights agree with known biological processes such as interferon-β (IFN-β) stimulation or maladaptive regeneration after kidney injury.

## Methods

### spVIPES inference

Given N=2 scRNA-seq matrices $x_{1}$ and $x_{2}$ (raw counts) with dimensions $C_{1}\times G_{1}$ and $C_{2}\times G_{2}$ (where $C_{i}$ denotes the number of cells and $G_{i}$ denotes the number of genes for group i), we estimate latent variables $z_{1}∼q_{\phi_{1}}\left(z|x_{1},s_{1}\right)$ and $z_{2}∼q_{\phi_{2}}\left(z|x_{2},s_{2}\right)$ where $q_{\phi_{1}}$ and $q_{\phi_{2}}$ are variational distributions parameterized by neural networks $\phi_{1}$ and $\phi_{2}$. Both distributions follow a prior $p\left(z\right)∼N\left(0,I\right)$. Variables $s_{1}$ and $s_{2}$ are one-hot-encoded batch indices. We assume that the latent representation $z_{i}$ for group i factorizes into private ($z_{i}^{p}$) and shared ($z_{i}^{s}$) components. To ensure $z^{s}$ encodes shared information between groups, we employ a PoE framework. Since our model takes unpaired samples and features, we need some level of supervision to obtain a meaningful joint distribution. Given $C_{1}$- and $C_{2}$-dimensional vectors $l_{1}$ and $l_{2}$, let M be the total number of unique classes (i.e., cell-type labels). For any class c, let $x_{c,i}$ denote the subset of samples in matrix $x_{i}$ belonging to class c. Then, for each class c=1,…,M:

$$q\left(z_{c}^{\mathrm{PoE}}|D_{c}\right)\propto N\left(z_{c}^{s};\;0,\;I\right)\prod_{i:c\in l_{i}}q\left(z_{c}^{s}|x_{c,i}\right).$$ (1)

where $D_{c}$ represents the set of all input subsets associated with class c (i.e., $D_{c}={\left\{x_{c,i}\right\}}_{i:c\in l_{i}}$), and the product runs over all groups i where class c is observed. $q\left(z_{c}^{s}|x_{c,i}\right)$ is the approximate posterior for class c over the shared latent space given the input subset $x_{c,i}$.

The combined mean $\mu_{c}^{\mathrm{PoE}}$ and variance ${\left(\sigma_{c}^{\mathrm{PoE}}\right)}^{2}$ for the PoE distribution are computed as:

$$\mu_{c}^{\mathrm{PoE}}={\left(1+\sum_{i:c\in l_{i}}\frac{1}{{\left(\sigma_{c,i}\right)}^{2}}\right)}^{-1}\sum_{i:c\in l_{i}}\frac{\mu_{c,i}}{{\left(\sigma_{c,i}\right)}^{2}},$$ (2)

$${\left(\sigma_{c}^{\mathrm{PoE}}\right)}^{2}={\left(1+\sum_{i:c\in l_{i}}\frac{1}{{\left(\sigma_{c,i}\right)}^{2}}\right)}^{-1}$$ (3)

Here, $\mu_{c,i}$ and ${\left(\sigma_{c,i}\right)}^{2}$ are the mean and variance of the approximate posterior from group i. We concatenate the PoE distributions to form an ordered tensor for group i: $z_{i}^{\mathrm{PoE}}=\left[q_{i}\left(z_{1}^{\mathrm{PoE}}\right),\ldots ,q_{i}\left(z_{M}^{\mathrm{PoE}}\right)\right]$.

### spVIPES generative process

We model each cell $x_{n}$ in a scRNA-seq data matrix x with a Negative Binomial with mixture parameters. Specifically, each entry $x_{n}$ arises from private and shared factors of variation, encoded by $z^{p}$ and $z^{s}$, respectively. Given $z_{n}^{s}$, $z_{n}^{p}$, $z_{n}^{\mathrm{ps}}$, $s_{n}$, and the observed RNA library size $\tau_{n}$ (calculated as the sum of RNA counts for each entry $x_{n}$), we define the following generative process:

$$\pi_{n}=f_{\pi }\left(z_{n}^{\mathrm{ps}},\;s_{n}\right)$$ (4)

$$\rho_{n}=f_{\rho }\left(z_{n}^{p},\;s_{n}\right)$$ (5)

$$\gamma_{n}=f_{\gamma }\left(z_{n}^{\mathrm{PoE}},\;s_{n}\right)$$ (6)

$$w_{\mathrm{ng}}|z_{n},s_{n}∼\text{Bernoulli}\left(\pi_{\mathrm{ng}}\right)$$ (7)

$$x_{n}|z_{n},s_{n},\tau_{n}∼\mathrm{Negative Binomial}\left(w_{\mathrm{ng}}\tau_{n}\gamma_{\mathrm{ng}}+\left(1-w_{\mathrm{ng}}\right)\tau_{i}\rho_{\mathrm{ng}},\;\theta_{g}\right)$$ (8)

where $\gamma_{n}$ and $\rho_{n}$ are the scale parameters of the shared and private latent variables, respectively. The mixture weights w are sampled from a Bernoulli distribution with parameter π given $z^{\mathrm{ps}}$ and s. The scale parameters are then regularized by the observed library size τ and multiplied by the mixture weights w to obtain the rate of the negative binomial. The inverse dispersion of the distribution is represented by the parameter θ.

### Learning objective

Given the intractable nature of our model’s true posterior $p\left(x_{i}|z_{i}^{\mathrm{ps}},s_{i}\right)$, we use variational inference [29] to estimate an approximate posterior of the form $q_{\phi_{i}}\left(z^{\mathrm{ps}}|x_{i},s_{i}\right)$. After inference, we obtain a negative binomial with mixture parameters $p_{\pi_{i}\rho_{i}\gamma_{i}}\left(x_{i}|z_{i}^{p},z_{i}^{\mathrm{PoE}},s_{i},\tau_{i}\right)$. Generally, the evidence lower bound [30] maximizes $E_{q\left(z|x\right)}\left[\log\left(p\left(x|z\right)\right)\right]$ and minimizes the Kullback–Leibler (KL) divergence $\mathrm{KL}\left(q_{\phi_{i}}\left(z|x\right),p\left(z\right)\right)$. For an arbitrary number of groups i=1,…,N, we minimize the following objective:

$$\begin{array}{c} \begin{array}{c} \sum_{i=1}^{N}E_{q_{\phi_{i}}\left(z^{p}|x_{i},\;s_{i}\right),q_{\phi_{i}}\left(z^{\mathrm{PoE}}|x_{i},\;s_{i}\right)}\left[\log\left(p_{\pi_{i}\rho_{i}\gamma_{i}}\left(x_{i}|\;z_{i}^{p},\;z_{i}^{\mathrm{PoE}},\;s_{i},\;\tau_{i}\right)\right)\right]\\ \;-\mathrm{KL}\left(q_{\phi_{i}}\left(z^{\mathrm{PoE}}|x_{i}\right),\;p\left(z^{\mathrm{PoE}}\right)\right)-\mathrm{KL}\left(q_{\phi_{i}}\left(z^{p}|x_{i}\right),\;p\left(z^{p}\right)\right) \end{array} \end{array}$$ (9)

### Unsupervised PoE via optimal transport

While the supervised approach described above requires high-quality cell-type annotations across all groups, in many scenarios, such labels may be unavailable, incomplete, or difficult to harmonize across datasets. To address this limitation, we developed an unsupervised variant of spVIPES that leverages optimal transport (OT) to identify cell correspondences across groups without relying on explicit supervision.

The unsupervised approach replaces the label-based PoE with a transport plan-guided PoE. Given the learned shared representations $z_{1}^{s}$ and $z_{2}^{s}$ from the 2 groups, we compute an OT plan $T\in {\mathbb{R}}^{C_{1}\times C_{2}}$ that minimizes the distance between their empirical distributions. If the two datasets have matched features, this corresponds to solving the classic Wasserstein optimal transport problem:

$$T^{*}=\arg\min_{T\in \Pi \left(\mu_{1},\;\mu_{2}\right)}\sum_{i=1}^{C_{1}}\sum_{j=1}^{C_{2}}T_{\mathrm{ij}}\cdot d\left(z_{1,i}^{s},\;z_{2,j}^{s}\right)$$ (10)

where $\Pi \left(\mu_{1},\;\mu_{2}\right)$ denotes the set of transport plans with marginals $\mu_{1}$ and $\mu_{2}$, and $d\left(\cdot ,\;\cdot \right)$ is the Euclidean distance in the shared latent space. The transport plan $T^{*}$ provides soft cell-to-cell correspondences, where $T_{\mathrm{ij}}^{*}$ represents the probability of pairing cell i from group 1 with cell j from group 2. We utilize this plan to construct the joint posterior via 2 distinct matching strategies:

i. Cell-level Matching (Hard 1-to-1 Pairing): In this strategy, we enforce a strict 1-to-1 correspondence by selecting the maximum transport weight for each cell. For each cell i in group 1, we identify its best match $j^{*}$ in group 2 as:

$$j^{*}=\arg\max_{j}T_{\mathrm{ij}}^{*}$$ (11)

The PoE is then computed using the parameters of the approximate posteriors for the specific pair $\left(i,\;j^{*}\right)$. Specifically, if cell i has posterior parameters $\left(\mu_{1,i},\;\sigma_{1,i}^{2}\right)$ and cell $j^{*}$ has $\left(\mu_{2,j^{*}},\;\sigma_{2,j^{*}}^{2}\right)$, the joint PoE distribution is derived directly from these 2 sets of parameters using Eqs. (2) and (3). This approach, which is the default in spVIPES-OT, effectively treats the inferred pairings as pseudo-ground-truth labels.

ii. Cluster-level Matching (Soft Weighted Aggregation): Alternatively, we implemented a soft matching strategy where the transport plan is used to aggregate statistics from the opposing group. Instead of selecting a single partner, we compute a weighted average of the posterior parameters from group 2 based on the row-normalized transport plan $\bar{T}$. For cell i in group 1, the matching parameters ${\hat{\mu }}_{2\to 1,i}$ are computed as:

$${\hat{\mu }}_{2\to 1,i}=\sum_{j=1}^{C_{2}}{\bar{T}}_{\mathrm{ij}}\mu_{2,j}$$ (12)

The PoE is then computed between the local parameters $\left(\mu_{1,i},\;\sigma_{1,i}^{2}\right)$ and the aggregated parameters $\left({\hat{\mu }}_{2\to 1,i},\;{\hat{\sigma }}_{2\to 1,i}^{2}\right)$.

### Network architecture

The set of parameters for our inference ($\phi_{i}$) and generative process (i.e., $\pi_{i}$, $\rho_{i}$, and $\gamma_{i}$) are learned using encoder and decoder neural networks, respectively. The encoder networks ($f_{\phi_{i}}$) take as input the expression values of a cell and output the parameters of an approximate posterior distribution (mean and variance in our case). The decoder networks (i.e., $f_{\pi_{i}},f_{\rho_{i}},\;\text{and}\;f_{\gamma_{i}}$) take as input the sampled latent space (i.e., $z^{\mathrm{ps}},\;z^{p},\;\text{and}\;z^{\mathrm{PoE}}$) and output the different parameters of our generative model.

The encoder neural network has 2 consecutive hidden layers with 128 nodes. Both are followed by ReLU activation functions. The output of the last hidden layer is then passed through a dropout layer. Its output is used as input for 2 different networks with batch normalization that learn the parameters of $q\left(z^{\mathrm{ps}}|x,\;s\right)$, which in our case are the mean and variance parameters.

The generative module of spVIPES consists of 2 decoder neural networks, one for each latent component (i.e., private and shared) that take as input $\left(z^{p},\;s\right)$ and $\left(z^{\mathrm{PoE}},\;s\right)$, respectively. Both networks use the same parameters and lack hidden layers. We apply the *Softmax* function to the output of each network to obtain the scale parameters of the private and shared components of the negative binomial mixture. The mixture weights w are learned using a neural network with a single hidden layer with 256 nodes, ReLU activation, dropout, and batch normalization layers. We reinject z,s into the hidden nodes before obtaining the full-dimensional weights.

### Decoder factor loadings

To facilitate biological interpretation of the learned latent variables, we extract factor loadings from the linear decoder networks, similar to the approach used in LDVAE [19]. Since our generative model utilizes batch normalization in the decoder, the effective contribution of each gene to a latent factor depends on both the linear weight and the batch normalization scaling.

For a given decoder (shared or private), let W be the weight matrix of the linear layer (linking latent dimensions to genes), and let γ, $\sigma^{2}$, and ε be the scaling factor, running variance, and numerical stability constant of the subsequent batch normalization layer, respectively. We compute the corrected loading matrix $W_{\text{loadings}}$ as:

$$W_{\text{loadings}}=\mathrm{diag}\left(\frac{\gamma }{\sqrt{\sigma^{2}+\varepsilon }}\right)\cdot W$$ (13)

These loadings represent the magnitude of the contribution of a latent variable to a gene’s expression, conditional on the corresponding decoder path being active (i.e., subject to the mixing parameter π). A high absolute value in $W_{\text{loadings}}$ indicates that the gene is strongly driven by that specific latent factor.

### Model training

In the supervised variant of spVIPES, to learn a meaningful shared representation that is not biased toward the most frequent label, a balanced training scheme is needed. To this end, we implement a weighted data loader that ensures that each mini-batch of data contains the biggest set of labels possible given the user-defined batch size.

Given the set of labels L where $L_{i}$ represents the *i*th label, the frequency $f\left(L_{i}\right)$ of occurrence of label $L_{i}$ in the dataset, and a cell $c_{j}$ associated with label $L_{i}$ [denoted as $c_{j}\left(L_{i}\right)$], we assign a weight $w\left(c_{j}\right)$ to each cell $c_{j}$ for sampling $w\left(c_{j}\right)=\frac{1}{f\left(c_{j}\left(L_{i}\right)\right)}$.

When training spVIPES-OT, the model relies on standard random sampling to construct the mini-batches. Both model’s parameters are updated after each mini-batch according to the loss function defined in Eq. (7). Similar to other VAE-based methods [6,31], we use the Adam [32] optimizer with weight decay to update the network’s parameters. To prevent posterior collapse (i.e., the posterior and prior distributions are identical; hence, the encoded representation is noninformative), we employ KL-annealing during training.

### Latent space linear probing

To quantitatively evaluate the disentanglement of biological signals between the shared and private subspaces, we trained linear classifiers on the learned latent representations. We utilized a Ridge classifier (*sklearn.linear_model.RidgeClassifier*) to predict the simulation’s ground-truth labels (i.e., cell types and gene programs) from the latent coordinates. For the shared latent space, we evaluated the model’s ability to predict cell-type identity (which should be conserved) versus group-specific gene programs (which should be removed). Conversely, for the private latent space, we evaluated the ability to predict gene programs versus cell types. We performed 5-fold cross-validation for each task and reported the mean classification accuracy and standard deviation.

### Evaluation metrics

For each of the latent variables obtained, neighbors and the UMAP representation were computed with Scanpy [33] *preprocessing.neighbors()* and *tools.umap()* functions with default parameters. To quantitatively compare the quality of the embeddings, we computed cell-type mixing (i.e., shared variation) and gene program (i.e., private variation) separation metrics using scib-metrics [34] *Benchmark* module. Overall scores for each of the latent spaces are computed as a weighted average of Batch correction metrics (0.4) and Biological conservation metrics (0.6). The resulting metrics were min–max scaled. For the simulation experiment, to obtain a joint score of private and shared embeddings, a weighted average of each respective overall score (i.e., shared overall score, private 1 overall score, and private 2 overall score) was computed. Specifically we used 0.5, 0.25, and 0.25 for the shared overall score, first private overall score, and second private overall score, respectively.

### Simulated dataset experiment

For our simulations, we used Splatter [35] v1.22.0. The first simulation (i.e., shared factors of variation) was obtained using *splatSimulate* with 50,000 cells and 2,000 genes using the *method* parameter set to *groups*. We split these cells into 5 groups. We then simulated an extra 500 genes (i.e., private factors of variation) with the parameters *de.facLoc* and *lib.loc* set to 0.85 and 6, respectively. We split the cells into 4 groups. Two of these groups are considered to be from group 1 (total of 25,000 cells), and the other 2 groups are considered to be from group 2 (total of 25,000 cells). We normalized and visualized the cells using scater [36] *logNormCounts* and *runUMAP* on the full-dimensional data.

We ran spVIPES with the 2 groups’ raw counts as input. We obtained 7 shared and 5 private dimensions for each group after training for 160 (default, based on number of input cells) epochs. We used default dropout and learning rates (0.1 and 0.001, respectively). For comparisons, we ran MultigroupVI with 10 shared and 7 private dimensions. Due to dependency conflicts preventing the installation of the MultigroupVI package, we were unable to retrain the model to match the optimal dimensionality configuration found for spVIPES. We trained the model for 170 epochs with a Wasserstein penalty of 1. We used default dropout and learning rates. Additionally, ContrastiveVI was run with default parameters using dataset 1 and dataset 2 as both the background and target datasets. Similarly, scDisInFact was run with default parameters.

### Multispecies integration experiment

We downloaded the zebrafish and frog datasets from their respective repositories (GSE112294 [37] and GSE113074 [38], respectively). Cell annotations were obtained from the SAMap paper [23]. We kept cells from both datasets with matching developmental stages and removed genes with no available symbols. As in the SAMap publication, we removed cells from the following zebrafish cell types: Apoptotic-like, Apoptotic-like 2, Epiphysis, and Nanog-high. Additionally, we also removed frog cells with the following annotations: Heart and Olfactory placode.

We ran spVIPES with 10 shared and 35 private dimensions for the default number of epochs (175) and retrieved the shared embeddings. Additionally, we used the developmental stage as the *batch_key* parameter in spVIPES and the cell-type annotations as the *label* parameter. For comparisons, MultigroupVI was run with 30 shared and 30 private dimensions. Due to dependency conflicts preventing the installation of the MultigroupVI package, we were unable to retrain the model to match the specific dimensionality configuration used for spVIPES. scVI was run using default latent dimensions and trained for 175 epochs using the species of origin as the *batch_key* parameter and the developmental stage as an additional categorical covariate. To run scANVI, we randomly set 20% of the annotations from the dataset to *Unknown*. We then trained the model imported from scVI for 20 extra epochs with the *unknown_category* set to the *Unknown* and the *label* set to the cell-type annotations. For the principal component analysis (PCA), we used Scanpy’s *tools.pca()* with default parameters (i.e., 50 PCs). We ran LIGER with default parameters and used the nonmatching features to aid the integration process. Finally, SATURN was run with default parameters using protein embeddings from the ESM-2 model [39]. To perform Gene Ontology (GO) enrichment on each dataset, we used g:Profiler [40] with default parameters.

### Acute kidney injury experiment

We downloaded the aligned data matrix from GSE180420 [41]. We considered samples IRI_short_1d, IRI_short_3d, and IRI_short_14d as part of group ischemia–reperfusion injury short (IRI-short). Samples IRI_long_1d, IRI_long_3d, and IRI_long_14d were grouped into ischemia–reperfusion injury long (IRI-long). We trained spVIPES for default epochs (106) with default hyperparameters. We set the sample of origin and the available cell-type annotations as the *batch_key* and *label* parameters of spVIPES. We retrieved 19 shared and 5 private dimensions for each group. Differential expression tests were performed using *tools.rank_genes_groups()* using Wilcoxon’s rank sum test.

### Stimulated PBMC experiment

We used the data generated by Kang et al. [42]. Train and validation datasets were downloaded from scGen’s [43] reproducibility repository. These were merged to train spVIPES, as it performs a 90:10 split (train:validation) by default. We trained the model for default epochs (250) considering control and stimulation as the 2 groups used as input to spVIPES. We retrieved 10 shared and 5 private dimensions per group. Graph-based clustering was performed using *tools.leiden()* with a resolution of 0.2. Differential expression tests were performed using Wilcoxon’s rank sum test.

## Results

### spVIPES model overview

**Fig. 1.**
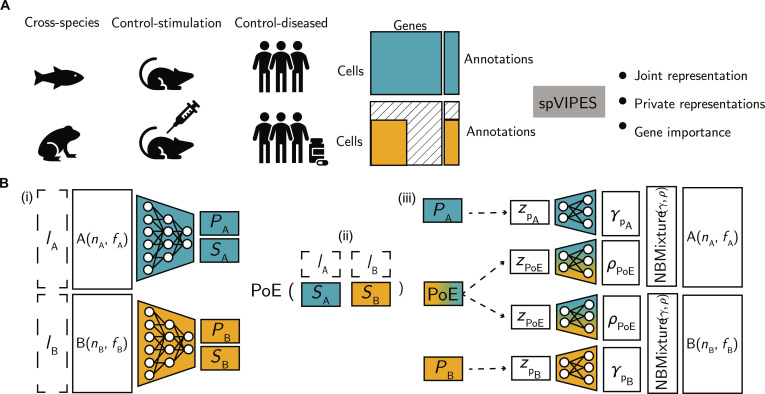
spVIPES model overview. (A) spVIPES takes as input 2 scRNA-seq matrices with raw UMI counts, with optional cell annotations and covariate vectors. For downstream tasks, spVIPES outputs a joint representation learned from both matrices, private representations for each of the groups present, and the importance of each of the genes present in each group for both the joint and the private representations. (B) spVIPES contains 3 main modules: (a) inference module, with networks for each of the groups; (b) PoE module; and (c) generative module, with networks for each of the groups and latent variables. The inference and generative modules are identical between groups. Dashed arrows indicate sampling from a distribution using the reparameterization trick. Dashed boxes represent the optional annotation vectors.

spVIPES posits that under a multigroup description of a cell’s identity, its latent representation can be split into a shared component and as many private components as groups are present. To this end, we use a probabilistic latent variable model [44] that can operate in both supervised and unsupervised modes to represent cells in private and shared latent spaces while accounting for technical differences between and within groups. The input to spVIPES is 2 scRNA-seq matrices with an arbitrary number of cells and genes (Fig. 1A). These matrices should contain unnormalized RNA unique molecular identifier (UMI) counts. For the supervised version, a vector representing each cell’s identity (i.e., cell types) is required, necessitating a previous step of label harmonization that can be achieved using existing pipelines in Seurat or Scanpy or other cell-type matching methods (e.g., MetaNeighbor [45]). The unsupervised version leverages OT to identify cell correspondences without requiring cell-type annotations. Optionally, spVIPES takes an additional categorical vector representing batches or other covariates of interest that could drive technical differences within the groups. spVIPES outputs: (a) the joint latent representation, (b) each group’s private representation, and (c) the weights from each group’s decoder network.

All 3 components are learned using a double VAE architecture (Fig. 1B), in which the distribution parameters for the latent spaces and the generative processes are optimized simultaneously. Using 2 different inference networks, we can model an arbitrary number of nonmatching input features. To learn a shared representation from these features that accounts for the shared biological processes between the groups, we employ a PoE framework. This framework takes the learned parameters from each inference as input and produces a combined set of parameters as its output. However, achieving a meaningful joint distribution derived from the PoE is challenging, especially considering the random selection of cells during training. To tackle this challenge, the supervised version incorporates cell-type information, computing a PoE using the location and scale parameters of each group for each cell type, while the unsupervised version uses OT to identify cell correspondences and applies PoE to the matched cell pairs. The sampled joint (i.e., shared) and private representation are then used as input to 2 independent decoder networks that learn the parameters of a generative mixture model similar to totalVI [31]. We model each UMI count as a mixture of private and shared components, with the mixture weights learned during training. By treating each latent representation independently and using linear decoders as in LDVAE [19], we can obtain disentangled gene weights for both the private and shared latent variables.

#### Performance on a simulated dataset

**Fig. 2.**
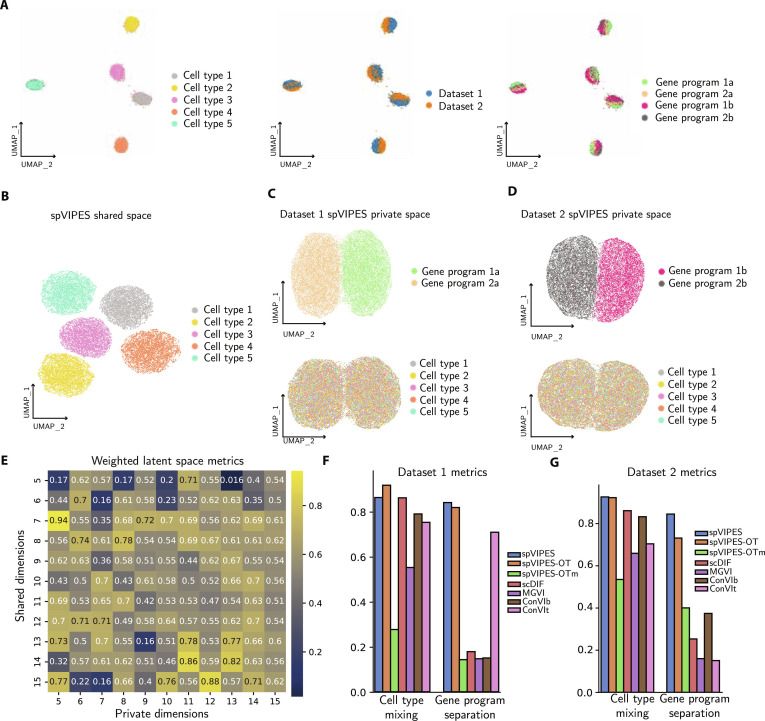
spVIPES performance on a simulated dataset. (A) UMAP representations of the 50 PCs from the dataset generated using Splatter. Cells are colored by cell type, dataset, and gene program. (B) UMAP representations of the shared embedding generated by spVIPES. (C and D) UMAP representations of the private embeddings for dataset 1 (C) and dataset 2 (D). Cells are colored by dataset-specific gene programs (top) or cell type (bottom). (E) Heatmap of latent space integration metrics for 64 combinations of shared and private dimensions. (F and G) Bar plots of cell-type mixing and gene program separation scores for the private spVIPES, spVIPES-OT, spVIPES-OTm (cluster-level matching), MultigroupVI (MGVI), and scDisInFact (scDIF) embeddings as well as salient ContrastiveVI embeddings using the dataset as background (ConVIb) or target (ConVIt) for dataset 1 (F) and dataset 2 (G).

To test whether spVIPES can capture global structure (i.e., cell types) in the shared space and more nuanced differences in each private space, we simulated a dataset using Splatter [35], following the setup proposed by Weinberger et al. [15] (Fig. 2A, refer to Methods for details).

Private spaces should learn group-specific structure (i.e., gene programs in our simulated dataset) while learning no structure shared among groups (i.e., cell types). Qualitatively, the shared and private latent spaces learned by spVIPES capture shared and dataset-specific variation, respectively, but the private spaces do not capture shared variation for any of the datasets (Fig. 2B to D).

To study how the model behaves with different latent dimension choices, we trained spVIPES using combinations of shared and private dimensions (between 5 and 15). We retrieved the private and shared embeddings and computed their respective batch correction, biological conservation metrics, and overall metrics as implemented in scib-metrics [34] (Fig. S1 and Tables S1 to S3). To understand which combinations had the best mixture of private and shared embeddings, we computed a weighted average of shared (0.5) and private (0.25 and 0.25 for dataset 1 and dataset 2, respectively) latent space over all metrics. We observed that increasing or decreasing the dimensionality of the private space did not yield a clear improvement in representation learning. Instead, performance fluctuated between combinations (Fig. 2E). Based on these results, we identified an optimal configuration of 7 shared and 5 private dimensions for this dataset. We performed the same grid search for the unsupervised OT variant (using 1-to-1 matching) and similarly observed no clear trend, identifying an optimal configuration of 6 shared and 6 private dimensions (Tables S4 to S6).

Given that the supervised version of spVIPES requires labels for training, we next sought to understand how label mismatches between the datasets affect the quality of our private and shared embeddings. First, we randomly switched the labels from both datasets in an increasing number of cells and evaluated the quality of the embeddings using scib-metrics. The ability of our shared embedding to separate cell types and mix dataset-specific information (i.e., gene programs) is directly dependent on the quality of the input labels; as the percentage of mismatched labels increases, the quality of the shared representation degrades. However, for each of the private embeddings, we observed that both gene program separation and cell-type mixing scores remained largely independent of the level of mismatch between the datasets (Fig. S2A). We further investigated the embeddings generated by spVIPES when one or more cell types are unique to one of the 2 datasets. We removed an increasing number of cell types from dataset 1 and quantitatively assessed the overlap of these unique cell types in the shared embedding. We computed the median distance between all matching cell types between dataset 1 and dataset 2 in each scenario. We computed the median distance from the unique cell types from dataset 2 (i.e., missing cell types from dataset 1) to all other cell types in dataset 1. We observed that unique cell types do not overlap with other cells in the shared latent space, even when multiple cell types are removed. In addition, matching cell types show a lower median distance between datasets, indicating a higher overlap in the high dimensional space (Fig. S2B). Additionally, we assessed the sensitivity of the unsupervised model to the regularization strength (ε) of the OT solver. We found that higher regularization values yielded the highest quality embeddings for both the shared and private spaces (Fig. S2C).

Next, to quantitatively determine the effectiveness of spVIPES in separating group-specific variation in its private spaces, we compared cell-type mixing and group-specific gene program separation metrics between the embeddings generated by spVIPES, MultigroupVI, scDisInFact, and ContrastiveVI. Additionally, we evaluated the performance of our unsupervised OT variant (spVIPES-OT), as well as a cluster-level matching variant (spVIPES-OTm), to assess whether comparable results could be achieved without requiring cell-type annotations. We considered cell types as batches to “integrate” and the group-specific gene programs as cell types that we aim to separate. We tried several hyperparameters, latent dimensions, and training epochs for MultigroupVI but could not replicate the results reported in the original publication using our simulated dataset. Instead, MultigroupVI fails to learn group-specific structure and mistakenly learns more shared variation than spVIPES. Moreover, ContrastiveVI is highly dependent on the choice of background and target dataset when learning its private (salient) space. Finally, scDisInFact performs well on cell-type mixing metrics but cannot properly disentangle dataset-specific information in its private space(s). Notably, spVIPES-OT achieved performance comparable to the supervised version across both cell-type mixing and gene program separation metrics, demonstrating that meaningful disentanglement can be achieved without requiring labeled data (Fig. 2F and G). In contrast, the cluster-level matching strategy (spVIPES-OTm) performed worse than both the supervised and cell-level OT variants. To further confirm this disentanglement, we trained linear classifiers on the latent representations. The shared spaces of both spVIPES and spVIPES-OT achieved near-perfect accuracy in predicting cell types (1.00 ± 0.00 and 0.99 ± 0.00, respectively) but low accuracy for gene programs (0.26 ± 0.00 and 0.29 ± 0.00). Conversely, the private spaces accurately predicted gene programs (0.82 ± 0.00 for spVIPES; 0.92 ± 0.00 for spVIPES-OT) while failing to distinguish cell types (0.24 ± 0.00 and 0.21 ± 0.00, respectively).

### spVIPES identifies cells and genes associated with stimulation

Next, we tested spVIPES with a published human peripheral blood mononuclear cells (PBMCs) dataset [46]. This study stimulated PBMCs with IFN-β, a cytokine that drives genome-wide changes in immune cell transcription. We expect spVIPES to identify the stimulation signal only in cells from the stimulated samples. Moreover, the genes responding to IFN-β should have high weights in the stimulation group’s private dimensions.

**Fig. 3.**
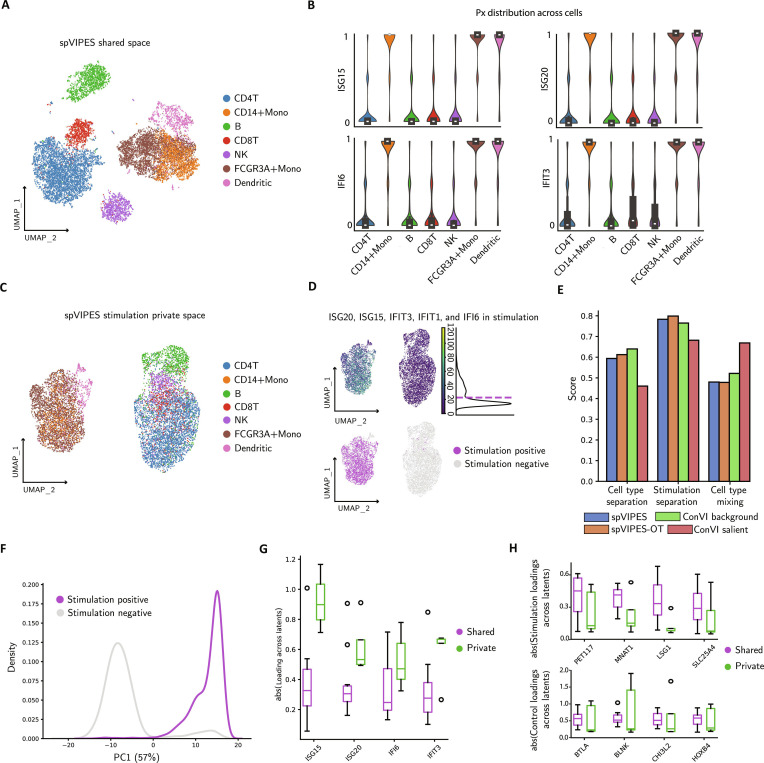
spVIPES identifies cells and genes associated with stimulation. (A) UMAP representation of the shared latent space between control and stimulated PBMCs. Cells are colored by cell type. (B) Violin plots showing the distribution of the mixing parameter (π) for IFN-β response genes across cell types. A binary shift indicates the model routing signal through private latents for responding populations (monocytes and dendritic cells). (C) UMAP representation of spVIPES’ private space for the stimulation group. Cells are colored by cell type. (D) (Top) UMAP representation of spVIPES’ private space for the stimulation group colored by *ISG20*, *ISG15*, *IFIT3*, and *IFI6* mean counts. The mean count distribution is shown alongside the legend, with the threshold chosen to split the populations shown in the purple dashed line. (Bottom) UMAP representation of spVIPES’ private space for the stimulation group. Cells are colored if they are enriched for stimulation signal. Otherwise, they are colored gray. (E) Barplots of the integration metrics scores for spVIPES, spVIPES-OT, and ContrastiveVI (ConVI) private (salient in the case of ConVI) and shared (background in the case of ConVI) embeddings. (F) Distribution of stimulation-positive and stimulation-negative cells along the first principal component (PC1). The variance explained by this component is shown in parentheses. (G) Box plots of the absolute weights of known stimulation genes (*ISG20*, *ISG15*, *IFIT3*, and *IFI6*) across shared and private latent dimensions. (H) Box plots of the genes with the highest absolute weight difference ($|W_{\text{shared}}|-|W_{\text{private}}|$) in the stimulation (top) and control (bottom) groups, highlighting genes strongly driven by the shared space.

We applied spVIPES specifying the control and stimulated samples as the 2 input groups. The obtained shared embedding correctly captures the major immune types in both stimulated and control samples (Fig. 3A). To verify if the model correctly routes the stimulation signal to the private space only for relevant cell types, we inspected the mixture parameter (π) of the Negative Binomial distribution for genes known to respond to IFN-β (*ISG20*, *ISG15*, *IFIT3*, and *IFI6*). We observed that cell types known to respond to stimulation (i.e., CD14^+^ monocytes, FCGR3A^+^ monocytes, and dendritic cells) exhibited a distinct binary behavior compared to nonresponding types. This indicates that spVIPES consistently utilizes the private latent dimensions to model expression in these specific populations, effectively “switching on” the private decoder pathway for the stimulation response (Fig. 3B).

To confirm that the private space captures the biological response, we retrieved the stimulation group’s private embedding. After dimensionality reduction, we observed cell-type mixing (e.g., among T-cell subtypes and B cells) while the global structure was driven by the stimulation status (Fig. 3C). To validate this, we visualized the median count distribution of genes associated with stimulation across all cells and binarized the population into stimulation-positive and stimulation-negative groups (Fig. 3D). Upon projection, we confirmed that the private space separates cells based on their stimulation signature rather than cell-type information.

To quantify the quality of these representations, we compared spVIPES (supervised and unsupervised OT variants) against ContrastiveVI. We treated the control dataset as background and the stimulated one as target for ContrastiveVI, comparing its background space to our shared space, and its salient space to our private space. In the shared space, spVIPES showed marginally lower cell-type separation compared to ContrastiveVI’s background model (Fig. 3E). However, in the private space, both spVIPES variants outperformed ContrastiveVI in separating the stimulation signal, which is the primary objective of the private embedding. Interestingly, while ContrastiveVI’s salient space maximized cell-type mixing, spVIPES retained some cell-type structure, likely reflecting that the magnitude of the IFN-β response varies by cell type. spVIPES-OT achieved the highest stimulation separation score but struggled with cell-type separation in the shared space, likely due to imperfections in the unsupervised transport plan. The separation of stimulation-positive and -negative cells in spVIPES’ private space was further confirmed by their distinct distribution along the first principal component (Fig. 3F).

Finally, we utilized the linear decoders of spVIPES to validate the biological interpretability of the latent dimensions. We compared the absolute weights of the stimulation genes (*ISG20*, *ISG15*, *IFIT3*, and *IFI6*) and observed that they were consistently higher in the private dimensions than in the shared dimensions (Fig. 3G). In contrast, we identified genes with the strongest preference for shared space (highest delta between shared and private weights). In the stimulation group, the top shared-driven genes included *PET117*, *MNAT1*, *LSG1*, and *SLC25A4*. These genes are involved in fundamental cellular processes such as mitochondrial function and ribosome biogenesis. In the control group, the top shared-driven genes included *BTLA*, *BLNK*, and *HOXB4* (Fig. 3H). *BTLA* and *BLNK* are critical B-cell identity markers, while *HOXB4* is a key hematopoietic regulator. Their strong weighting in the shared space confirms that spVIPES correctly captures stable cell-type information and fundamental housekeeping processes in the shared latent variables, while separating the transient stimulation response to the private space.

### spVIPES improves integration of distant species

**Fig. 4.**
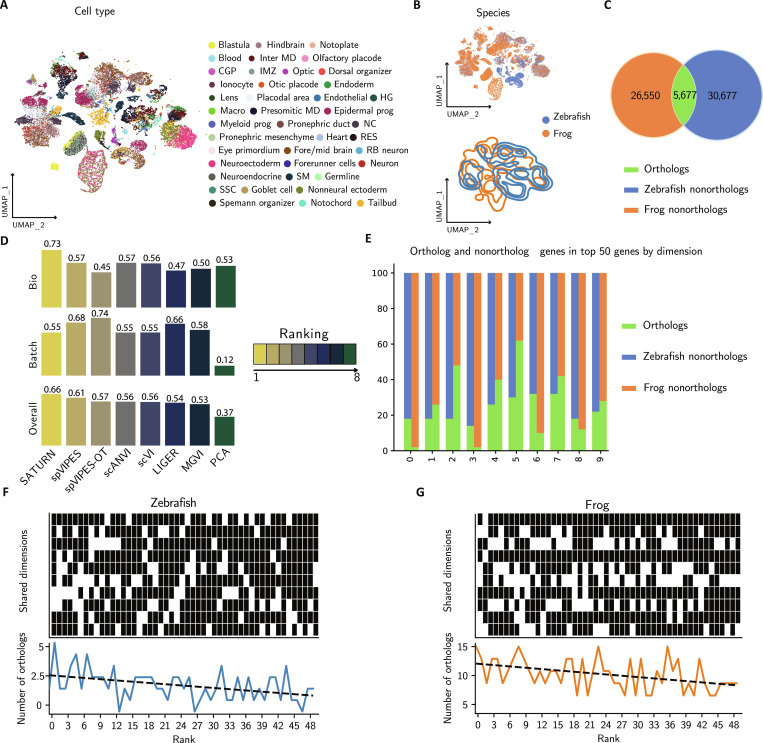
spVIPES improves integration of distant species. (A) UMAP representation of the shared latent space between zebrafish and frog. Cells are colored by cell type. (B) UMAP representation (top) and density plot (bottom) of the shared embedding generated by spVIPES. Cells are colored by species of origin. (C) Venn diagram showing the overlap (orthologous genes) between zebrafish and frog. The number on the outer circles represents nonorthologous genes. (D) Benchmarking of spVIPES’ and spVIPES-OT’s shared latent space against SATURN, MultigroupVI, scVI, scANVI, LIGER, and PCA using batch correction metrics (Batch) and biological conservation metrics (Bio). Models are ordered by overall score. (E) Distribution of orthologs and nonortholog genes in the top 20 genes by loading in the shared latent space. (F and G) Binary matrix plots with nonortholog (black) and ortholog (white) genes at each shared dimensions’ top 50 ranks for frog (F) and zebrafish (G) shared dimensions. The total sum of orthologs per rank is visualized below each matrix. RB, Rohon-Beard; MD, mesoderm; IMZ, involuting marginal zone; Macro, macrophage; HG, heart gland; NC, neural crest; RES, rare epidermal subtypes; SSC, small secretory cells; SM, skeletal muscle.

One of the key features in spVIPES is its ability to integrate datasets with nonmatching features. Transcriptomics datasets from different species are integrated based on their shared feature space, inferred from ortholog relationships between the genes [47]. Incorporating more complex relationships between genes across different species has been shown to improve cell-type matching [24]. We applied spVIPES to 2 developmental scRNA-seq datasets of *Danio rerio* (zebrafish from now on) and *Xenopus tropicalis* (frog from now on) [37,38]. Both datasets consist of whole-body single-cell transcriptomics at different embryonic stages. We found that, despite the complexity of these data, spVIPES could retain the common cellular identities in its shared space (Fig. 4A), while effectively mixing both species within the shared embedding (Fig. 4B).

We then compared spVIPES’ shared latent space to alternative modeling approaches. We defined 1-to-1 orthologs between the 2 species using BioMart [48] to obtain a common feature space. After concatenation, only 5,677 ortholog genes remained (Fig. 4C). We benchmarked spVIPES and its unsupervised variant, spVIPES-OT, against the dedicated cross-species integration tool SATURN [25], as well as general-purpose integration methods including scVI [6], scANVI [49], MultigroupVI [15], and linear models such as LIGER [50] and PCA. Cell-type separation and batch (species) mixing metrics were calculated with scib-metrics [34]. While SATURN achieved the highest overall performance, both spVIPES and spVIPES-OT outperformed all other general-purpose modeling approaches in both biological conservation and batch correction metrics (Fig. 4D). Notably, spVIPES-OT achieved competitive performance without requiring matched cell-type labels, demonstrating the robustness of the OT initialization.

We assessed if nonortholog genes were deemed important drivers of cellular identity in spVIPES’ shared latent space. We observed that over half of the top 50 genes with the biggest loading across dimensions were nonortholog genes (Fig. 4E). To further understand the importance of nonortholog genes in our model, we visualized the contribution of ortholog and nonortholog genes to each dimension’s top 50 ranks. We did not observe a clear prioritization of ortholog or nonortholog genes among the top ranks (Fig. 4F and G), suggesting that they are equally important in our model.

Finally, to biologically validate the shared latent space, we analyzed the functional enrichment of the top orthologous genes driving the shared representation. We extracted the top 100 orthologs with the highest global loading magnitude (L2 norm across shared dimensions) for both zebrafish and frog and performed GO enrichment analysis. We found a significant overlap in key developmental terms, including “cell differentiation” and “cellular developmental process”, confirming that the shared space captures conserved biological programs driving embryogenesis across species despite the evolutionary distance (Table S7).

### spVIPES accurately identifies genes associated with maladaptive regeneration after acute kidney injury

Chronic kidney disease (CKD) is a complex condition with multiple contributing factors. However, it is widely recognized that kidney injury can cause lasting fibrosis, which can ultimately lead to the development of CKD [51,52]. To demonstrate spVIPES’ latent space interpretability, we applied our model to an scRNA-seq acute kidney injury (AKI) dataset in mice [41]. In this study, the authors compared 2 different models of AKI to a matched control at 1, 3, and 14 d after injury: (a) a short-induced injury characterized by immediate repair (IRI-short) and (b) a longer injury followed by maladaptive repair (IRI-long). Our goal with spVIPES was to identify the maladaptive signature enriched in the private space corresponding to the long injury model compared to the short injury model.

**Fig. 5.**
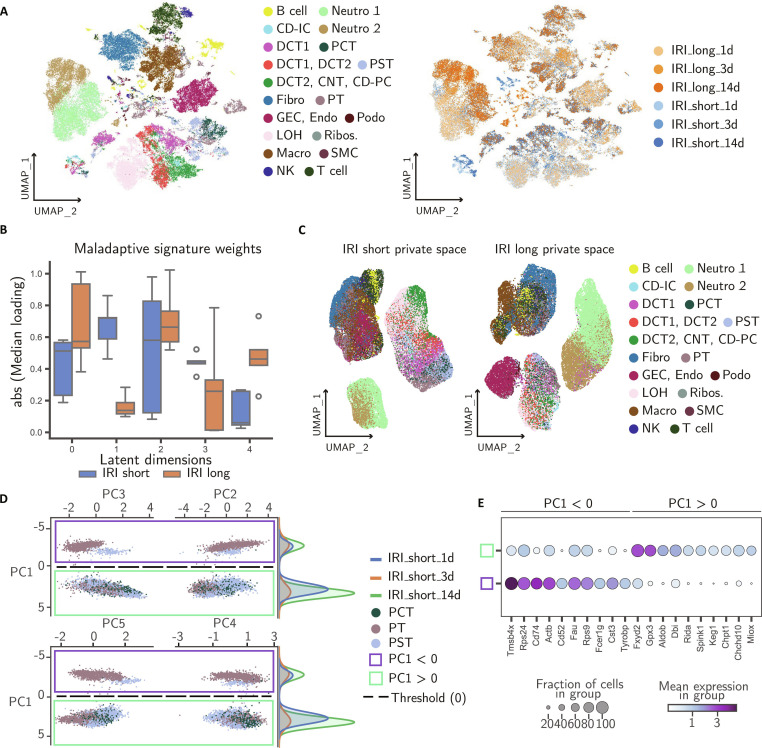
spVIPES accurately identifies genes associated with maladaptive regeneration and injury-repair populations of cells. (A) UMAP representation of the shared latent space between IRI-long and IRI-short samples. Cells are colored by cell type and sample of origin. (B) UMAP representations of the private latent space of IRI-short (left) and IRI-long (right) samples. (C) Box plot of the median loading of *Fxyd5*, *Il1b*, *Cxcl2*, *Ccl3*, and *Tyrobp* across private dimensions in IRI-short and IRI-long. (D) PCA plots of spVIPES IRI-short private embedding for PT, PST, and PCT cells. Cells are colored by cell type. The dashed line (PC1 = 0) splits the cells into the Failed Repair population (PC1 < 0) and the Successful Repair population (PC1 > 0). The distribution of cells belonging to the different IRI-short samples (1, 3, and 14 days after injury) is shown along the PC1 axis (duplicate view). (E) Normalized expression dot plot of the top 10 differentially expressed genes for the Failed Repair (PC1 < 0) and Successful Repair (PC1 > 0) populations. CD-IC, collecting duct intercalated cell; DCT, distal convoluted tubule; PCT, proximal convoluted tubule; PST, proximal straight tubule; CNT, connecting tubule; GEC, glomerular endothelial cell; LOH, loop of Henle; Podo, podocytes; PT, proximal tubule; CD-PC, collecting duct principal cell; SMC, smooth muscle cell; Macro, macrophage; NK, natural killer; Ribos., ribosome-enriched population; Neutro, neutrophil; Fibro, fibroblast.

To this end, we considered IRI-long and -short as the 2 groups used as input to spVIPES. After dimensionality reduction of spVIPES’ shared space, we observed strong mixing across samples and separation between major cell types (Fig. 5A). For example, DCT1, DCT2, CNT, and CD-PC are cell types of the distal part of the nephron, with overlapping molecular functions such as water reabsorption and urine concentration [53]. spVIPES’ shared space correctly learns this similarity. In addition, we observed strong mixing between cell types in each of the group’s private spaces (Fig. 5B). One of the key differences between IRI-long and -short samples found by the authors was the expression of a maladaptive signature (i.e., *Fxyd5*, *Il1b*, *Cxcl2*, *Ccl3*, and *Tyrobp*) in IRI-long proximal tubules. To assess whether our model can learn the importance of the maladaptive signature in IRI-long samples compared to short ones, we examined the private latent dimensions obtained by spVIPES. We retrieved the weights corresponding to each group’s private decoder. We observed that specific private dimensions (0, 2, and 4) in the IRI-long group exhibited higher absolute median loadings for the maladaptive signature compared to the IRI-short group, while other dimensions showed the opposite trend (Fig. 5C). This suggests that spVIPES selectively segregates the maladaptive biological signal into distinct latent factors within the private space.

#### spVIPES identifies successful and failed repair outcomes in proximal tubules

To further study PTs in the IRI-short group, we retrieved its private embedding. After performing PCA on the embedding space, we observed that the first principal component (PC1) separates PTs into 2 distinct transcriptional states that do not strictly correspond to time points, but rather to divergent repair outcomes. The positive PC1 axis is enriched for cells from day 14, while the negative PC1 axis contains an approximately equal composition of cells from days 1, 3, and 14 (Fig. 5D). Differential expression analysis revealed that these populations likely represent successful versus failed repair states.

The day 14-enriched population (positive PC1) is characterized by high expression of *Aldob*, a key glycolytic/gluconeogenic enzyme marking mature PT identity, alongside a robust antioxidant module including *Gpx3*, *Prdx5*, and the aldo-keto reductases *Akr1a1* and *Akr1c21* (Fig. 5E). This profile aligns with the “successfully repaired” PT state described in recent literature [54,55], where cells regain differentiated function while maintaining a metabolic shield against oxidative stress. The specific expression of *Akr1a1*, which detoxifies reactive aldehydes and protects against injury via metabolic regulation [56], suggests that these cells have fully recovered a healthy, resilient phenotype by day 14.

In contrast, the population with mixed contributions from days 1, 3, and 14 (negative PC1) expresses markers of acute injury and inflammation, including *Tmsb4x*, *Cst3*, and the immune-interaction module *Cd74*, *Fcer1g*, and *Tyrobp* (Fig. 5E). *Cd74* serves as a receptor for macrophage migration inhibitory factor (MIF), driving pro-inflammatory NF-κB signaling in tubular epithelium [57]. The presence of day 1 and day 3 cells in this cluster represents the canonical acute injury phase. However, the persistence of day 14 cells in this state suggests a failure to transition to the repaired *Aldob*-high state. These cells resemble the “Failed Repair” proximal tubule cells (FR-PTCs) described by Kirita et al. [55], which remain undifferentiated and pro-inflammatory, potentially acting as seeds for fibrosis and maladaptive repair even in the short-injury model. Thus, spVIPES’ private space effectively disentangles the bifurcating fate of PTs: the majority that successfully redifferentiate and a minority that remain trapped in a maladaptive, inflammatory state.

## Discussion

spVIPES is a deep probabilistic framework designed to encode grouped single-cell RNA-seq data into shared and private factors of variation. Traditional nonlinear deep generative models [6,49] assume that a cell’s high-dimensional gene expression results from a flat set of latent variables. This assumption holds in single-group scenarios (i.e., the control cohort in a control-disease study), in which a common intermediate biological representation should be present despite within-group technical differences. However, the application of these models beyond single groups lacks generative motivation; both control and disease cells are assumed to be generated from the same set of latent variables, which can be problematic in complex scenarios. We introduce a model that treats a cell’s count as a mixture of both shared and private factors of variation between groups of single-cell RNA-seq samples. Moreover, we employ a flexible inference that encodes shared factors of variation using a PoE framework.

We showed that spVIPES improves upon previous models attempting to disentangle structured latent features (i.e., private and shared representations). Additionally, spVIPES improves the integration of largely nonoverlapping feature spaces, such as in zebrafish and frog developmental datasets [37,38]. Although specialized cross-species integration methods [25,26] can achieve high integration scores, they typically map data to a single embedding, obscuring species-specific biological signals. We further demonstrated spVIPES’ ability to capture nuanced variations previously overlooked by identifying a novel injury-associated population of neutrophils in an AKI mouse dataset [41]. Beyond characterizing cell types in the shared and private latent spaces, spVIPES can be used to inspect how different genes contribute to each latent variable using its linear generative module. Using a published dataset consisting of PBMCs with and without IFN-β stimulation, we showed that stimulation-associated genes have a shifted loading in the stimulation-specific variables compared to the shared ones.

spVIPES will be beneficial in single-cell studies where traditional models struggle to disentangle complex biological variation. The ability to obtain private and shared representations is particularly important in studies involving nonoverlapping feature spaces and sample–sample relationships with biological relevance. Our approach is also instrumental when building or expanding cell atlases. When using other nonlinear models, the number of features that remain after integration decreases as more datasets are included due to the use of different technologies, processing pipelines, or species of origin. This is aggravated when considering the use of both targeted and untargeted approaches, which yield different number of features by design. spVIPES can address these issues, successfully integrating and batch correcting these datasets in its shared space.

Limitations have to be considered. The supervised version of spVIPES requires matched labels for the input groups, necessitating a prior step of harmonization. This requirement for matched cell-type labels can limit application in complex real-world settings involving species-specific cell types or one-to-many correspondences. Such harmonization can typically be achieved using existing pipelines in Seurat or Scanpy or other cell-type matching methods (e.g., MetaNeighbor [45]). While our unsupervised OT variant addresses this limitation by removing the need for annotations, it may exhibit reduced performance in cell-type separation compared to the supervised version, particularly when the transport matrix contains suboptimal pairings. Nevertheless, we found this approach to be robust to the regularization strength of the transport plan, and our use of 1-to-1 cell matching helps maintain population structure even in the absence of labels. The selection of latent space dimensionality is dataset-dependent, so we recommend that users applying spVIPES to new datasets optimize these dimensions. Typically, tasks involving large between-group differences (e.g., cross-species) may benefit from larger private dimensions to absorb group-specific variation, whereas tasks with subtle perturbations (e.g., stimulation) may benefit from constrained private dimensions to prevent information leakage. We also note that while spVIPES disentangles sources of variation, we do not explicitly regularize the model to prevent information leakage between subspaces. Due to the inherent symmetries in mixture models, this lack of explicit constraints implies that the specific routing of signals to private versus shared paths can be subject to stochastic variation across random initializations. Finally, in its current state, spVIPES does not consider multimodal nuances in its generative model, such as those introduced by totalVI or peakVI [31,58]. However, our PoE-based inference offers flexibility, making it feasible to adapt spVIPES for multimodal datasets once these nuances are integrated. Although we demonstrated spVIPES using 2 groups as input, the extension to multiple groupings is straightforward.

## Data Availability

All datasets used in this manuscript are publicly available. Accession numbers are provided for zebrafish (GSE112294), frog (GSE113074), and kidney AKI (GSE180420) datasets. We downloaded the PBMC stimulation dataset from https://github.com/theislab/scgen-reproducibility. The simulated data generated in this manuscript are available at https://zenodo.org/records/10070301.

## References

[1] Stubbington MJT, Rozenblatt-Rosen O, Regev A, Teichmann SA. Single-cell transcriptomics to explore the immune system in health and disease. Science. 2017;358(6359):58–63. 28983043 10.1126/science.aan6828 PMC5654495

[2] Park J, Shrestha R, Qiu C, Kondo A, Huang S, Werth M, Li M, Barasch J, Susztãk K. Single-cell transcriptomics of the mouse kidney reveals potential cellular targets of kidney disease. Science. 2018;360(6390):758–763. 29622724 10.1126/science.aar2131 PMC6188645

[3] Kirita Y, Haojia W, Uchimura K, Wilson PC, Humphreys BD. Cell profiling of mouse acute kidney injury reveals conserved cellular responses to injury. Proc Natl Acad Sci USA. 2020;117(27):15874–15883. 32571916 10.1073/pnas.2005477117 PMC7355049

[4] Ding J, Condon A, Shah SP. Interpretable dimensionality reduction of single cell transcriptome data with deep generative models. Nat Commun. 2018;9(1):2002. 29784946 10.1038/s41467-018-04368-5 PMC5962608

[5] Eraslan G, Simon LM, Mircea M, Mueller NS, Theis FJ. Single-cell RNA-seq denoising using a deep count autoencoder. Nat Commun. 2019;10(1):390. 30674886 10.1038/s41467-018-07931-2 PMC6344535

[6] Lopez R, Regier J, Cole MB, Jordan MI, Yosef N. Deep generative modeling for single-cell transcriptomics. Nat Methods. 2018;15(12):1053–1058. 30504886 10.1038/s41592-018-0229-2 PMC6289068

[7] Novella-Rausell C, Grudniewska M, Peters DJM, Mahfouz A. A comprehensive mouse kidney atlas enables rare cell population characterization and robust marker discovery. iScience. 2023;26(6): Article 106877. 37275529 10.1016/j.isci.2023.106877 PMC10238935

[8] Swamy VS, Fufa TD, Hufnagel RB, McGaughey DM. Building the mega single-cell transcriptome ocular meta-atlas. GigaScience. 2021;10(10): Article giab061. 34651173 10.1093/gigascience/giab061 PMC8514335

[9] Herpelinck T, Ory L, Nasello G, Barzegari M, Bolander J, Luyten FP, Tylzanowski P, Geris L. An integrated single-cell atlas of the skeleton from development through adulthood. biorXiv. 2022. 10.1101/2022.03.14.484345 PMC12215971 40596066

[10] Zhu J-Y, Zhang R, Pathak D, Darrell T, Efros AA, Wang O, Shechtman E. Toward multimodal image-to-image translation. In: *Proceedings of the 31st International Conference on Neural Information Processing Systems*. Red Hook (NY): Curran Associates, Inc.; 2018. p. 465–476.

[11] Gonzalez-Garcia A, van de Weijer J, Bengio Y. Image-to-image translation for cross-domain disentanglement. In: *Proceedings of the 32nd International Conference on Neural Information Processing Systems*. Red Hook (NY): Curran Associates, Inc.; 2018. p. 1294–1305.

[12] Lee M, Pavlovic V. Private-shared disentangled multimodal VAE for learning of latent representations. In: *Proceedings of the IEEE/CVF Conference on Computer Vision and Pattern Recognition(CVPR) Workshops*. IEEE; 2021. p. 1692–1700.

[13] Mourragui SMC, Siefert JC, Reinders MJT, Loog M, Wessels LFA. Identifying commonalities between cell lines and tumors at the single cell level using Sobolev alignment of deep generative models. bioRxiv. 2022. 10.1101/2022.03.08.483431

[14] Weinberger E, Lin C, Lee S-I. Isolating salient variations of interest in single-cell data with contrastiveVI. *Nat Methods*. 2023;20(9):1336–1345. 10.1038/s41592-023-01955-3 37550579

[15] Weinberger E, Lin C, Lee D-H. Disentangling shared and group-specific variations in single-cell transcriptomics data with multigroupvi. *Nat Methods*. 2024;21:467476.

[16] Zhang Z, Zhao X, Bindra M, Qiu P, Zhang X. scDisInFact: Disentangled learning for integration and prediction of multi-batch multi-condition single-cell RNA-sequencing data. Nat Commun. 2024;15(1):912. 38291052 10.1038/s41467-024-45227-w PMC10827746

[17] Minoura K, Abe K, Nam H, Nishikawa H, Shimamura T. A mixture-of-experts deep generative model for integrated analysis of single-cell multiomics data. Cell Rep Methods. 2021;1(5): Article 100071. 35474667 10.1016/j.crmeth.2021.100071 PMC9017195

[18] Samuel K. Ainsworth, Nicholas J. Foti, Adrian K. C. Lee, and Emily B. Fox. oi-VAE: Output interpretable VAEs for nonlinear group factor analysis. In: Dy J, Krause A, editors. *Proceedings of the 35th International Conference on Machine Learning*, Vol. 80 of *Proceedings of Machine Learning Research*. PMLR; 2018, p. 119–128.

[19] Svensson V, Gayoso A, Yosef N, Pachter L. Interpretable factor models of single-cell rna-seq via variational autoencoders. Bioinformatics. 2020;36(11):3418–3421. 32176273 10.1093/bioinformatics/btaa169 PMC7267837

[20] Seninge L, Ioannis Anastopoulos H, Ding, and Joshua Stuart. Vega is an interpretable generative model for deciphering biological mechanisms in single-cell transcriptomic data. Nat Commun. 2021;12(1): Article 5639. 34584103 10.1038/s41467-021-26017-0 PMC8478947

[21] Choi Y, Li R, Quon G. Sivae: Interpretable deep generative models for single-cell transcriptomes. Genome Biol. 2023;24(1):31. 36803416 10.1186/s13059-023-02850-y PMC9940350

[22] Gabaldãn T, Koonin EV. Functional and evolutionary implications of gene orthology. Nature Review Genetics. 2013;14(5):360–366. 10.1038/nrg3456 PMC5877793 23552219

[23] Tarashansky AJ, Musser JM, Khariton M, Li P, Arendt D, Quake SR, Wang B. Mapping single-cell atlases throughout metazoa unravels cell type evolution. eLife. 2021;10: Article e66747. 33944782 10.7554/eLife.66747 PMC8139856

[24] Biharie A, Michielsen L, Reinders MT, Mahfouz A. Cell-type matching across species using protein embeddings. Bioinformatics. 2023;39:i404–i412. 37387141 10.1093/bioinformatics/btad248 PMC10311290

[25] Rosen Y, Brbic M, Roohani Y, Swanson K, Li Z, Leskovec J. Toward universal cell embeddings: Integrating single-cell RNA-seq datasets across species with saturn. Nat Methods. 2024;21:1492–1500. 38366243 10.1038/s41592-024-02191-z PMC11310084

[26] De Donno C, Hediyeh-Zadeh S, Moinfar AA, Wagenstetter M, Zappia L, Lotfollahi M, Theis FJ. Population-level integration of single-cell datasets enables multi-scale analysis across samples and conditions. Nat Methods. 2023;20:1683–1692. 37813989 10.1038/s41592-023-02035-2 PMC10630133

[27] Zhang Y et al. Icebear: An interpretable co-embedding method for single-cell multi-omics data integration. Genome Biol. 2025;26(1).

[28] Sun H, Haowen Q, Duan K, Wei D. Scmgcn: A multi-view graph convolutional network for cell type identification in scrna-seq data. Int J Mol Sci. 2024;25(4):2234. 38396909 10.3390/ijms25042234 PMC10889820

[29] Kingma DP, Welling M. Auto-encoding variational bayes. arXiv. 2013. 10.48550/arXiv.1312.6114

[30] Blei DM, Kucukelbir A, McAuliffe JD. Variational inference: A review for statisticians. J Am Stat Asscoc. 2017;112(518):859–877.

[31] Gayoso A, Steier Z, Lopez R, Regier J, Nazor KL, Streets A, Yosef N. Joint probabilistic modeling of single-cell multi-omic data with totalVI. Nat Methods. 2021;18(3):272–282. 33589839 10.1038/s41592-020-01050-x PMC7954949

[32] Kingma DP, Ba J. Adam: A method for stochastic optimization. arXiv. 2014. 10.48550/arXiv.1412.6980

[33] Wolf FA, Angerer P, Theis FJ. SCANPY: Large-scale single-cell gene expression data analysis. Genome Biol. 2018;19(1):15. 29409532 10.1186/s13059-017-1382-0 PMC5802054

[34] Luecken MD, Büttner M, Chaichoompu K, Danese A, Interlandi M, Mueller MF, Strobl DC, Zappia L, Dugas M, Colomé-Tatché M, et al. Benchmarking atlas-level data integration in single-cell genomics. Nat Methods. 2022;19(1):41–50. 34949812 10.1038/s41592-021-01336-8 PMC8748196

[35] Zappia L, Phipson B, Oshlack A. Splatter: Simulation of single-cell rna sequencing data. Genome Biol. 2017;18(1):174. 28899397 10.1186/s13059-017-1305-0 PMC5596896

[36] McCarthy DJ, Campbell KR, Lun ATL, Wills QF. Scater: Pre-processing, quality control, normalization and visualization of single-cell RNA-seq data in R. Bioinformatics. 2017;33(8):1179–1186. 28088763 10.1093/bioinformatics/btw777 PMC5408845

[37] Wagner DE, Weinreb C, Collins ZM, Briggs JA, Megason SG, Klein AM. Single-cell mapping of gene expression landscapes and lineage in the zebrafish embryo. Science. 2018;360(6392):981–987. 29700229 10.1126/science.aar4362 PMC6083445

[38] Briggs JA, Weinreb C, Wagner DE, Megason S, Peshkin L, Kirschner MW, Klein AM. The dynamics of gene expression in vertebrate embryogenesis at single-cell resolution. Science. 2018;360(6392): Article eaar5780. 29700227 10.1126/science.aar5780 PMC6038144

[39] Lin Z, Akin H, Rao R, Hie B, Zhu Z, Wenting L, Smetanin N, Verkuil R, Kabeli O, Shmueli Y, et al. Evolutionary-scale prediction of atomic-level protein structure with a language model. Science. 2023;379(6637):1123–1130. 36927031 10.1126/science.ade2574

[40] Kolberg L, Raudvere U, Kuzmin I, Vilo J, Peterson H. G:Profiler: A web server for functional enrichment analysis and conversions of gene lists(2023 update). Nucleic Acids Res. 2023;51(W1):W207–W212. 37144459 10.1093/nar/gkad347 PMC10320099

[41] Balzer MS, Doke T, Yang Y-W, Aldridge DL, Hu H, Mai H, Mukhi D, Ma Z, Shrestha R, Palmer MB, et al. Single-cell analysis highlights differences in human and mouse kidney development and injury response. JCI Insight. 2022;7(13): Article e153572. 10.1038/s41467-022-31772-9 PMC9276703 35821371

[42] Kang HM, Subramaniam M, Targ S, Nguyen M, Maliskova L, McCarthy E, Wan E, Wong S, Byrnes L, Lanata CM, et al. Multiplexed droplet single-cell RNA-sequencing using natural genetic variation. Nat Biotechnol. 2017;36(1):89–94. 29227470 10.1038/nbt.4042 PMC5784859

[43] Mohammad Lotfollahi F, Wolf A, Theis FJ. scGen predicts single-cell perturbation responses. Nat Methods. 2019;16(8):715–721. 31363220 10.1038/s41592-019-0494-8

[44] Hofmann T. Unsupervised learning by probabilistic latent semantic analysis. Mach Learn. 2001;42(1/2):177–196.

[45] Crow M, Paul A, Sara Ballouz Z, Huang J, Gillis J. Characterizing the replicability of cell types defined by single cell RNA-sequencing data using MetaNeighbor. Nat Commun. 2018;9(1):884. 29491377 10.1038/s41467-018-03282-0 PMC5830442

[46] Kang HM, Subramaniam M, Targ S, Nguyen M, Maliskova L, Carthy EM, Wan E, Wong S, Byrnes L, Lanata CM, et al. Multiplexed droplet single-cell RNA-sequencing using natural genetic variation. Nat Biotechnol. 2018;36(1):89–94. 29227470 10.1038/nbt.4042 PMC5784859

[47] Gautam P, Hamashima K, Chen Y, Zeng Y, Makovoz B, Parikh BH, Lee HY, Lau KA, Su X, Wong RCB, et al. Multi-species single-cell transcriptomic data integration via conserved gene co-expression patterns. Nat Commun. 2023;14:5675. 34584087 10.1038/s41467-021-25968-8 PMC8478974

[48] Smedley D, Haider S, Ballester B, Holland R, London D, Thorisson G, Kasprzyk A. Biomart—Biological queries made easy. BMC Genomics. 2009;10:22. 19144180 10.1186/1471-2164-10-22 PMC2649164

[49] Chenling X, Lopez R, Mehlman E, Regier J, Michael J I, Yosef N. Probabilistic harmonization and annotation of single-cell transcriptomics data with deep generative models. Mol Syst Biol. 2021;17(1): Article e9620. 33491336 10.15252/msb.20209620 PMC7829634

[50] Liu J, Gao C, Sodicoff J, Kozareva V, Macosko EZ, Welch JD. Jointly defining cell types from multiple single-cell datasets using liger. Nat Protoc. 2020;15(11):3632–3662. 33046898 10.1038/s41596-020-0391-8 PMC8132955

[51] Kalantar-Zadeh K, Jafar TH, Nitsch D, Neuen BL, Perkovic V. Chronic kidney disease. Lancet. 2021;398(10302):786–802. 34175022 10.1016/S0140-6736(21)00519-5

[52] Humphreys BD. Mechanisms of renal fibrosis. Annu Rev Physiol. 2018;80:309–326. 29068765 10.1146/annurev-physiol-022516-034227

[53] Kokko JP. The role of the collecting duct in the regulation of excretion of sodium and other electrolytes. Kidney Int. 1987;31(2):606–610. 3550230 10.1038/ki.1987.41

[54] Gerhardt LMS, Liu J, Kaghazchi B, Cippà PE, McMahon AP. Single-nuclear transcriptomics reveals diversity of proximal tubule cell states in early acute kidney injury. J Am Soc Nephrol. 2021;32(6):1293–1311. 34183416 10.1073/pnas.2026684118 PMC8271768

[55] Kirita Y, Wu H, Uchimura K, Willson PC, Humphreys BD. Comprehensive profiling of single-cell transcriptomes of the human kidney response to acute injury. Proc Natl Acad Sci USA. 2020;117(27):15874–15883. 32571916 10.1073/pnas.2005477117 PMC7355049

[56] Zhou HL, Zhang R, Anand P, Stomberski CT, Qian Z, Hausladen A, Wang L, Rhee EP, Parikh SM, Karumanchi SA, et al. Metabolic reprogramming by the S-nitroso-CoA reductase system protects against kidney injury. Nature. 2019;565(7737):96–100. 30487609 10.1038/s41586-018-0749-z PMC6318002

[57] Sanchez-Nino MD et al. Mif/cd74 signaling drives glomerular injury in lupus nephritis. J Am Soc Nephrol. 2013;24.

[58] Ashuach T, Reidenbach DA, Gayoso A, Yosef N. PeakVI: A deep generative model for single-cell chromatin accessibility analysis. Cell Rep Methods. 2022;2(3): Article 100182. 35475224 10.1016/j.crmeth.2022.100182 PMC9017241

