# Supplementary material for: Integrative Learning of Disentangled Representations from Single-Cell RNA-Sequencing Datasets
Source: Comput Struct Biotechnol J. 2026 Mar 16;35(1):0015. doi: 10.34133/csbj.0015 (PMC13068006; doi:10.34133/csbj.0015)
Supplement: Supplementary 1 — Figs. S1 and S2 Tables S1 to S7 [file csbj.0015.f1.zip › Supplementary Material.pdf]

1 Supplementary Figures

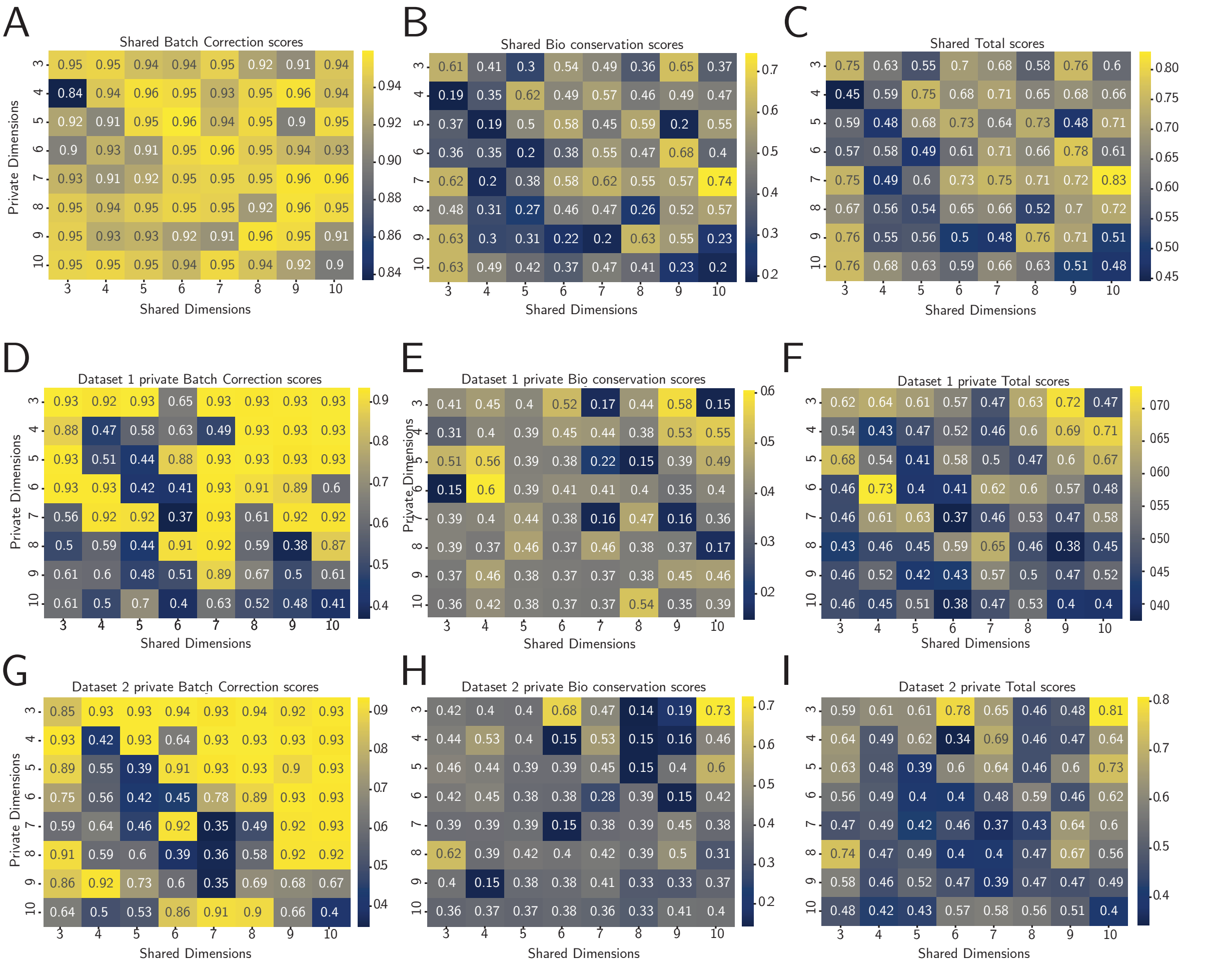

Supplementary Figure 1: *spVIPES latent space dimensionality evaluation* **A-C**. Heatmaps of shared latent space integration metrics for 64 combinations of shared and private dimensions, ranging from 3 to 10. Scores are computed as an average of batch correction metrics (A) biological conservation metrics (B) and a weighted average (0.4 and 0.6 for batch correction and biological conservation metrics) of the two (C). **D-F**. Heatmaps of dataset 1 private latent space integration metrics. Scores are computed as an average of batch correction metrics (D) biological conservation metrics (E) and a weighted average of the two (F). **G-I**. Heatmaps of dataset 2 private latent space integration metrics. Scores are computed as an average of batch correction metrics (D) biological conservation metrics (E) and a weighted average of the two (F).

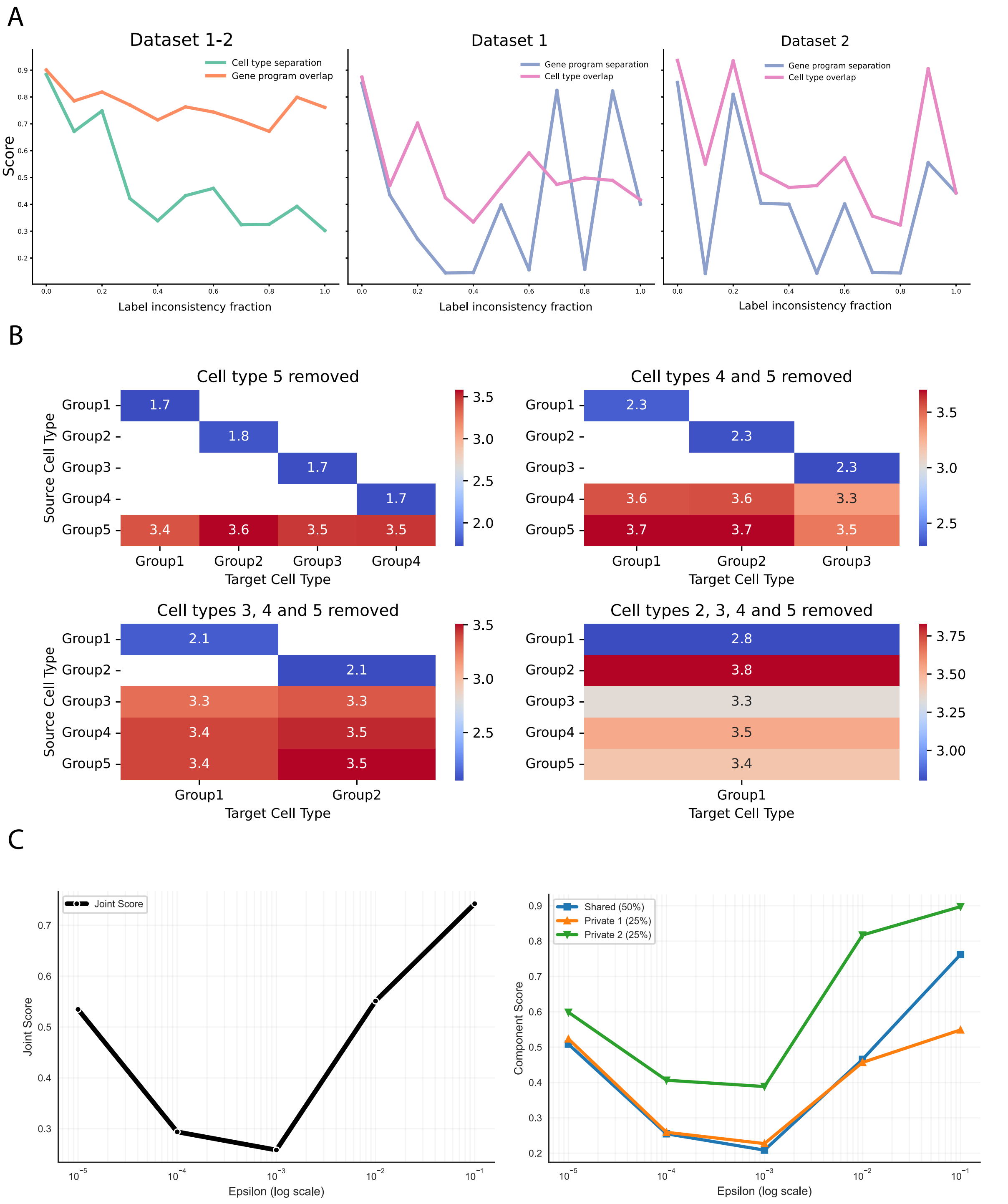

Supplementary Figure 2: *spVIPES latent space label mismatch evaluation* **A**. Lineplots of shared and private latent space integration metrics for different fractions of label mismatch. **B**. Heatmaps of the median distance between cell types from Dataset 1 and Dataset 2 at increasing number of missing cell types. For unique cell types (i.e., removed from Dataset 1) distances were computed for all other possible cell types in Dataset 1.

2 Supplementary Tables

Supplementary Tables 1–3. Benchmarking metrics for supervised spVIPES under varying dimensionality. Evaluation metrics computed using *scib-metrics* for the latent spaces generated by the supervised spVIPES model on the simulated dataset. Rows correspond to different combinations of shared and private latent dimensions. **Table 1** reports metrics for the shared latent space. **Table 2** and **Table 3** report metrics for the private latent spaces of Group 1 and Group 2, respectively. Columns include biological conservation scores (NMI, ARI, cLISI), batch correction scores (iLISI, ASW batch), and the overall weighted score.

Supplementary Tables 4–6. Benchmarking metrics for unsupervised spVIPES-OT under varying dimensionality. Evaluation metrics computed using *scib-metrics* for the latent spaces generated by the unsupervised variant (spVIPES-OT) on the simulated dataset. **Table 4** reports metrics for the shared latent space. **Table 5** and **Table 6** report metrics for the private latent spaces of Group 1 and Group 2, respectively. Columns follow the same metric definitions as Tables 1–3.

Supplementary Table 7. Gene Ontology enrichment analysis of shared space drivers in cross-species integration. Functional enrichment results (generated via g:Profiler) for the top 100 orthologous genes with the highest global loading magnitude in the shared latent space for the Zebrafish and Frog developmental datasets. Columns include the GO term name, GO ID, adjusted p-values for each species, and the conservation category.
